# Determination of an optimal dosing regimen for aspirin chemoprevention of 1,2-dimethylhydrazine-induced colon tumours in rats

**DOI:** 10.1038/sj.bjc.6690263

**Published:** 1999-04

**Authors:** C J Barnes, M Lee

**Affiliations:** Department of Medicine, University of Texas Health Science Center, Veterans Affairs Medical Center, San Antonio, Texas, 78284, USA

**Keywords:** aspirin, carcinogenesis, chemoprevention, colon, rat

## Abstract

In order to establish an optimal timing and duration of aspirin treatment in the chemoprevention of 1,2-dimethylhydrazine (DMH)-induced colon cancer in rats, colon tumours were induced using an established protocol and aspirin was given in the diet at 500 p.p.m. during various stages of colon carcinogenesis. Results indicate that only aspirin treatment throughout the entire carcinogenic period significantly reduced tumour incidence and volume whereas intermittent aspirin dosing increased tumour number and/or volume, suggesting that aspirin must be used for an extended period in order to gain any chemopreventive benefit. © 1999 Cancer Research Campaign


*British Journal of Cancer* (1999) **79**(11/12), 1646–1650
© 1999 Cancer Research Campaign
Article no. bjoc.1998.0263

Short communication

### Determination of an optimal dosing regimen for aspirin chemoprevention of 1,2-dimethylhydrazine-induced colon tumours in rats

**CJ Barnes and M Lee**

Department of Medicine, University of Texas Health Science Center, and Veterans Affairs Medical Center, San Antonio, Texas 78284, USA

**Summary** In order to establish an optimal timing and duration of aspirin treatment in the chemoprevention of 1,2-dimethylhydrazine (DMH)-induced colon cancer in rats, colon tumours were induced using an established protocol and aspirin was given in the diet at 500 p.p.m. during various stages of colon carcinogenesis. Results indicate that only aspirin treatment throughout the entire carcinogenic period significantly reduced tumour incidence and volume whereas intermittent aspirin dosing increased tumour number and/or volume, suggesting that aspirin must be used for an extended period in order to gain any chemopreventive benefit.

**Keywords:** aspirin; carcinogenesis; chemoprevention; colon; rat

As the second leading cause of cancer deaths, colorectal cancer is a significant source of morbidity and mortality in Western developed countries. Recent epidemiological studies have demonstrated a significant correlation between regular aspirin use and reduced colon cancer incidence and mortality (Giovannucci et al, 1995; Thun, 1996). Moreover, in patients with previous colon adenomas, aspirin use has been correlated with a reduced risk of adenoma recurrence (Greenberg et al, 1993). Aspirin has also been shown to suppress both the incidence and size of both carcinogen-induced rodent colon tumours (Craven and DeRubertis, 1992; Reddy et al, 1993) and spontaneous intestinal tumours in mice genetically predisposed to intestinal tumour formation (Barnes and Lee, 1998). However, the optimal dosage, timing and duration of aspirin chemoprevention have not been determined in animal models of colon carcinogenesis or in prospectively controlled human trials. The present study was designed to establish an optimal time period and duration of aspirin treatment in the chemoprevention of carcinogen-induced colon cancer in rats.

### MATERIALS AND METHODS

### Animals

Male Sprague-Dawley rats ($n$ = 208) were purchased at 2 months of age from Harlan Sprague-Dawley (Indianapolis, IN, USA) 2 weeks before the experiment. Rats had free access to food and water throughout these studies and were housed in wire-bottom cages to prevent coprophagy. All experimental procedures described below were approved by the Institutional Animal Care Program of the University of Texas Health Science Center (San Antonio, TX, USA).

*Received 24 March 1998*
*Accepted 28 May 1998*

*Correspondence to:* M Lee, Department of Medicine, Division of Digestive Diseases, University of Mississippi Medical Center, 2500 North State Street, Jackson, MS39216, USA

### Reagents

Dietary components for the AIN-76A diet were purchased from ICN (Costa Mesa, CA, USA). 1,2-Dimethylhydrazine (DMH), acetylsalicylic acid (aspirin) and serum salicylate assay kit were purchased from Sigma (St. Louis, MO, USA). All other chemicals were of the highest grade available and were obtained commercially. DMH vehicle contained 0.9% sodium chloride and 0.18% EDTA at pH 6.5. DMH and vehicle were prepared 1 h before injection.

### Experimental protocol

Upon receipt, rats were fed a modified AIN-76A diet (Reddy et al, 1993) made weekly in our laboratory as previously described (Barnes and Lee, 1998). After 2 weeks of acclimation and monitoring, rats were randomly divided into one of eight treatment groups as outlined below ($n$ = 8 in groups 1 and 2, $n$ = 32 in groups 3–8). During the various aspirin treatment periods specified below, groups 2 and 4–8 were switched from the standard modified AIN-76A diet to the same diet supplemented with 500 p.p.m. aspirin. Based on a previous report (Reddy et al, 1993), this aspirin dose should be sufficient to inhibit carcinogen-induced colon tumours without significantly affecting gains in body weight. Rats were weighed weekly throughout the study as a gross measure of toxicity. Moreover, rats were given a single s.c. injection of DMH (12 mg base $kg^{-1}$ b.w., groups 3–8) or DMH vehicle alone (4 ml $kg^{-1}$ b.w., groups 1 and 2) once a week for 8 weeks according to an established protocol for inducing rat colon cancer (Cameron et al, 1990). The treatment schedule was as follows:

| Group | DMH | Aspirin | Comments |
|---|---|---|---|
| 1 | None | None | Controls receiving DMH vehicle and no aspirin diet only |
| 2 | None | Entire 30 weeks | Controls for aspirin treatment and side-effects |
| 3 | Yes | None | Positive controls for tumour development |
| 4 | Yes | 1st 8 weeks only | Aspirin during colon cancer initiation period only |
| 5 | Yes | Weeks 9–16 only | Aspirin during promotional period only |
| 6 | Yes | Weeks 1–16 only | Aspirin during both initiation and promotional periods |
| 7 | Yes | Weeks 9–30 only | Aspirin during promotion and tumour development stages |
| 8 | Yes | Entire 30 weeks | Aspirin during the entire 30 week study |

After the 8-week initiation period, rats received no further injections and were killed 22 weeks later. Blood was collected by cardiac puncture for measurement of serum salicylate level (as an indicator of aspirin ingestion) using a commercial kit as previously described (Lee et al, 1994). Each colon was then immediately resected, rinsed with ice-cold phosphate-buffered saline (PBS, pH 7.4), and examined macroscopically to document tumour incidence, size and multiplicity by an observer blinded to treatment. Macroscopically visible tumours were excised and fixed in 10% buffered formalin. Histopathological examination was performed on haematoxylin and eosin stained, 4-μm-thick slide-mounted tumour sections according to the criteria of Sunter et al (1978).

### Statistical analysis

All statistical analyses were performed using PRISM (GraphPad Software, San Diego, CA, USA) statistical software. Data are expressed as absolute numbers or as means ± s.e.m. Differences in mean body weight per group, tumour multiplicity (defined as the number of tumours per tumour-bearing rat), tumour volume ($mm^3$), and serum salicylate concentration were determined using analysis of variance followed by Student–Newman–Keuls multiple range tests. Differences in tumour incidence (defined as the number of rats per group with tumours) were determined using Fisher's exact test. Statistical significance was accepted when $P<0.05$.

### RESULTS

To determine whether DMH treatment and/or 500 p.p.m. aspirin in the diet had any significant effect on weight gain, rats were weighed once a week throughout the experimental period. As shown in Figure 1, there were no significant differences in weight gain between various treatment groups. Also, blood was collected at the time of sacrifice in order to verify aspirin ingestion. Only rats consuming the aspirin diet at the end of the experimental period (i.e. groups 2, 7 and 8) had significant blood salicylate levels (Table 1), with no significant differences in the blood salicylate levels among these groups.

The effect of aspirin treatment during various stages of DMH-induced carcinogenesis on tumour incidence is summarized in Table 2. No tumours were noted in the DMH vehicle control rats (groups 1 and 2). The majority of DMH-induced tumours were found in the colon. Outside the colon, most tumours were located in the duodenum; however, one rat each in groups 3 and 4 had a zymbal gland tumour, and one rat each in groups 3 and 6 and two rats in group 5 had peritoneal metastases. Aspirin treatment throughout the carcinogenic period (i.e. group 8) was the only regimen which significantly reduced colon tumour incidence when compared with the DMH/no aspirin control group (i.e. group 3). Furthermore, group 8 was also the only experimental group which demonstrated a significant decrease in mean tumour volume when compared with the DMH/no aspirin control group 3 (Table 3). In contrast, intervention with aspirin during weeks 1–8 (i.e. group 4) or weeks 9–16 (i.e. group 5) caused a significant increase in mean colon tumour volume or total tumour volume when compared with the DMH/no aspirin control group (i.e. group 3). Finally, none of the treatment regimens used here significantly affected tumour multiplicity (group means, ranging from 1.5 ± 0.3 to 1.9 ± 0.2 tumours per tumour-bearing rat).

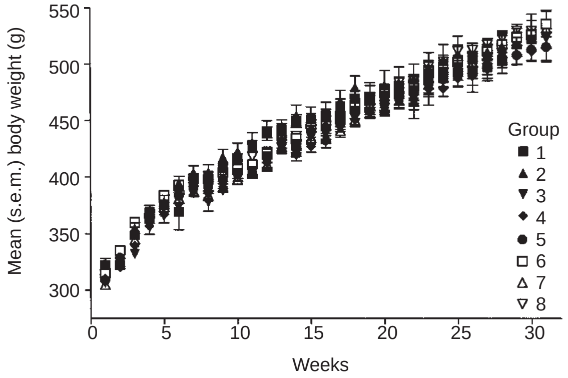

**Figure 1** Mean (s.e.m.) rat body weight in grams for each treatment group as defined in the Materials and Methods section. There were no significant differences in body weight gain between the various treatment groups

### DISCUSSION

The purpose of the present study was to determine an optimal time period and duration of aspirin treatment for the chemoprevention of colon cancer development in a rat model of carcinogen-induced colon cancer. Results indicate that only aspirin administration throughout the entire carcinogenic period significantly reduced colon tumour incidence and and tumour volume. Previous

**Table 1** Effect of aspirin intervention during different stages of DMH-induced carcinogenesis on blood salicylate levels in rats

| | Treatment group[a] | | | | | | | |
|---|---|---|---|---|---|---|---|---|
| | 1 | 2 | 3 | 4 | 5 | 6 | 7 | 8 |
| Aspirin duration (weeks) | N/A[b] | 1–30 | N/A | 1–8 | 9–16 | 1–16 | 9–30 | 1–30 |
| Blood salicylate | 0 ± 0[c] | 4.8 ± 0.2[d] | 0 ± 0[c] | 0 ± 0[c] | 0 ± 0[c] | 0 ± 0[c] | 6.0 ± 0.3[d] | 4.8 ± 0.2[d] |

[a]Rats received a subcutaneous injection of DMH vehicle (groups 1 and 2) or DMH (groups 3–8) once a week for 8 weeks, then no further injections for an additional 22 weeks; they were fed either a modified AIN-76A diet or the same diet supplemented with 500 p.p.m. aspirin as described in the Materials and methods section. Data are the means ± s.e.m. serum salicylate levels (mg $dl^{-1}$) in blood collected at the time of death using a commercial kit as previously described (Lee et al, 1994). [b]Rats consumed the no aspirin diet throughout the experimental period. [c]0 = Undetectable. [d]Significantly different from no aspirin control groups, $P<0.05$; no significant differences between groups with the same superscript.

© Cancer Research Campaign 1999

**Table 2** Effect of aspirin intervention during different stages of DMH-induced carcinogenesis on tumour incidence in rats

| **Treatment group**[a] | 3 | 4 | 5 | 6 | 7 | 8 |
|---|---|---|---|---|---|---|
| Aspirin duration (weeks) | N/A[b] | 1–8 | 9–16 | 1–16 | 9–30 | 1–30 |
| Tumour incidence (%) | | | | | | |
| Colon | 15 (47) | 10 (31) | 12 (38) | 14 (44) | 15 (47) | 5 (16)[d] |
| Small intestine/other[c] | 6 (19) | 7 (22) | 9 (28) | 5 (16) | 4 (13) | 6 (19) |
| Total | 19 (59) | 13 (41) | 17 (53) | 17 (53) | 15 (47) | 10 (31)[e] |

[a]Rats received a subcutaneous injection of DMH once a week for 8 weeks, then no further injections for an additional 22 weeks, and were fed either a modified AIN-76A diet or the same diet supplemented with 500 p.p.m. aspirin as described in the Materials and methods section. Tumour incidence was defined as the number of rats per group with tumours in each category (also as %), $n = 32$ per group. No tumours were noted in the control rats (groups 1 and 2). [b]Rats consumed the no aspirin diet throughout the experimental period. [c]Tumours outside the colon were found primarily in the duodenum, but tumours in the jejunum, zymbal gland tumours and peritoneal metastatic tumours also occurred. [d]Significantly different from groups 3, 6 and 7 in colon tumour incidence, $P < 0.05$. [e]Significantly different from group 3 in total tumour incidence, $P < 0.05$.

**Table 3** Effect of aspirin intervention during different stages of DMH-induced carcinogenesis on tumour volume in rats

| **Treatment group**[a] **Aspirin duration (weeks)** | **3** **N/A**[b] | **4** **1–8** | **5** **9–16** | **6** **1–16** | **7** **9–30** | **8** **1–30** |
|---|---|---|---|---|---|---|
| Tumour volume | | | | | | |
| Colon | 103.6 ± 17.3 | 132.2 ± 49.1 | 183.3 ± 20.2[d] | 97.4 ± 11.7 | 69.2 ± 15.5 | 22.7 ± 12.8[e] |
| SI/other[c] | 169.6 ± 24.7 | 530.8 ± 61.8[f] | 221.7 ± 45.7 | 250.3 ± 92.3 | 344.0 ± 116.6 | 74.1 ± 21.2 |
| Total | 124.7 ± 14.5 | 311.6 ± 48.1[d] | 192.4 ± 20.4[g] | 88.2 ± 9.8 | 130.2 ± 29.9 | 52.7 ± 12.4[h] |

[a]Rats received a subcutaneous injection of DMH once a week for 8 weeks, then no further injections for an additional 22 weeks; they were fed either a modified AIN-76A diet or the same diet supplemented with 500 p.p.m. aspirin as described in the Materials and methods section. Data are the means (s.e.m.) tumour volume ($mm^3$) in each group ($n = 32$ per group) and in each category. [b]Rats consumed the no aspirin diet throughout the experimental period. [c]Tumours outside the colon were found primarily in the duodenum, but tumours in the jejunum, zymbal gland tumours and peritoneal metastatic tumours also occurred. SI, small intestine. [d]Significantly different (i.e. higher) from all other groups in same tumour category, $P<0.05$. [e]Significantly different (i.e. lower) from groups 3–6 in colon tumour volume, $P<0.05$. [f]Significantly different (i.e. higher) from groups 3, 5, 6 and 8 in SI/other tumour volume, $P<0.05$. [g]Significantly different (i.e. higher) from groups 6 and 8 in total tumour volume, $P<0.05$. [h]Significantly different (i.e. lower) from groups 3–5 in total tumour volume, $P<0.05$.

research has demonstrated that continuous aspirin use suppressed spontaneous intestinal adenoma formation in a mouse model of familial adenomatous polyposis (Barnes and Lee, 1998). Also, aspirin inhibited carcinogen-induced rat colon tumours in a rat model when the drug was administered either throughout the carcinogenic process (Reddy et al, 1993) or given starting 1 week before and ending 1 week after carcinogen exposure (Craven and DeRubertis, 1992). Aspirin did not inhibit carcinogen-induced rat colon cancer when given just during the initiation period of carcinogenesis (Barnes et al, 1998*a*), when aspirin use started 1 week after carcinogen exposure (Craven and DeRubertis, 1992), or when aspirin was administered concomitantly with cholic acid during the promotion or the progression stages of a rodent colon cancer model (Pence et al, 1995). Moreover, previous research results using aberrant crypt foci (ACF) as a surrogate end point biomarker indicate that ACF formation was initially suppressed when aspirin was administered during the initiation period of carcinogenesis, but the initial aspirin-induced suppression is reversible upon cessation of aspirin treatment (Barnes et al, 1997). With one exception (Craven and DeRubertis, 1992), the present data are in agreement with previous research on aspirin chemoprevention of carcinogen-induced colon cancer in rodents. Importantly, the present report is the first demonstration using a single protocol of the necessity of extended aspirin administration for chemoprevention of colon cancer.

Although the precise mechanisms by which aspirin and other non-steroidal anti-inflammatory drugs (NSAIDs) exert their chemopreventive effects remain unclear, several lines of evidence suggest that this class of drugs inhibits gastrointestinal tumours via acetylation of cyclo-oxygenase (COX) isozymes and subsequent inhibition of intestinal prostaglandin production (Meade et al, 1993; Williams et al, 1997). First, the inducible isozyme COX-2 is elevated in human adenomas and adenocarcinomas (Eberhart et al, 1994; Kargman et al, 1995), in carcinogen-induced rodent tumours (DuBois et al, 1996), and in spontaneously occurring intestinal adenomas in two separate mouse models of familial adenomatous polyposis (FAP) (Oshima et al, 1996; Williams et al, 1996). Second, Oshima et al (1996) recently demonstrated that crossbreeding COX-2 gene knockout mice with $Apc^{\Delta 716}$ knockout mice (which normally develop hundreds of spontaneous intestinal polyps) dramatically reduced the number and size of intestinal polyps. Finally, various structurally unrelated NSAIDs, including aspirin, sulindac and the novel selective COX-2 inhibitors MF-tricyclic and SC-58635, have been shown to inhibit tumour formation in carcinogen-treated rodents (Craven and DeRubertis, 1992; Reddy et al, 1993, 1996; Rao et al, 1995) and in mouse models of FAP (Boolbol et al, 1996; Jacoby et al, 1996; Oshima et al, 1996; Chiu et al, 1997; Barnes and Lee, 1998).

Alternatively, aspirin and other NSAIDs may inhibit tumour formation via a prostaglandin independent pathway through

 *© Cancer Research Campaign 1999*

restoration of normal intestinal mucosal homeostasis, possibly by reducing cell proliferation (Barnes et al, 1995) and/or inducing apoptosis (Barnes et al, 1998*b*; Piazza et al, 1995; Morin et al, 1996). Additional mechanisms by which aspirin might also suppress colon tumour formation include acetylation of other protein(s) and/or other biological molecules (Abramson et al, 1985; Marnett, 1992), or via the production of 15-HETE from COX-2 after aspirin acetylation (Lecomte et al, 1994).

In the present study, aspirin was given at a moderate therapeutic dose (i.e. equivalent to a human antiarthritic dose of between four and eight 325-mg tablets per day); under this protocol, aspirin could work directly on DMH activation, on post-DMH activation events, or in both capacities depending on the specific treatment regimen. We have previously reported that aspirin given during the initiation period suppresses the formation of single aberrant crypts, but not the rate of ACF growth to multicrypt foci once formed (Barnes et al, 1997). Available data from our past research (Barnes et al, 1997, 1998*a*) and this report suggest that aspirin administration during the DMH induction process does not prevent molecular or cellular carcinogenic changes, but rather decreases the expression of this initiated phenotype. With intermittent aspirin intervention, the numbers and size of carcinogen-induced tumours are either similar to or actually surpass those parameters in the DMH/no aspirin treatment group. The phenomena of reversibility of a suppressive action has also been observed with sulindac, another NSAID, in patients with familial adenomatous polyposis (Giardiello, 1994). Likewise, a recent epidemiological study on aspirin use and colon cancer incidence and mortality stressed the need for many years of regular aspirin use in order to reap any chemopreventive benefit (Giovannucci et al, 1995). Taken together, available data suggest that continuous aspirin use for an extended period is needed to gain any chemopreventive benefits. The potential reversibility of NSAID chemoprevention and the need for extended aspirin use to reap a chemopreventive benefit must be considered when recommending aspirin use for colorectal cancer chemoprevention in humans.

### ACKNOWLEDGEMENTS

This work was supported in part by the San Antonio Cancer Institute and a National Institutes of Health Research Center Award (P30 CA 54174).

### ABBREVIATIONS

ACF, aberrant crypt foci; COX, cyclooxygenase; DMH, 1,2-dimethylhydrazine; FAP, familial adenomatous polyposis; NSAIDs, nonsteroidal antiinflammatory drugs.

### REFERENCES

Abramson S, Korchak H, Ludewig R, Edelson H, Haines K, Levin RI, Herman R, Rider L, Kimmel S and Weissman G (1985) Modes of action of aspirin-like drugs. *Proc Natl Acad Sci USA* **82**: 7227–7231

Barnes CJ and Lee M (1998) Chemoprevention of spontaneous intestinal adenomas in the $APC^{Min}$ mouse model with aspirin. *Gastroenterology* **114**: 873–877

Barnes CJ, Lee M, Hardman WE and Cameron IL (1995) Aspirin, age, and proximity to lymphoid nodules influence cell proliferation parameters in rat colonic crypts. *Cell Prolif* **28**: 59–71

Barnes CJ, Hardman WE, Cameron IL and Lee M (1997) Aspirin, but not sodium salicylate, indomethacin, or nabumetone, reversibly suppresses 1,2-dimethylhydrazine-induced colonic aberrant crypt foci in rats. *Dig Dis Sci* **42**: 920–926

Barnes CJ, Gauntt SA, Henderson LA, Yang Y, Ulmer RJ, Puleo-Scheppke BA, Stothoff AF, Hardman WE, Cameron IL and Lee M (1998*a*) Long term aspirin administration, but not intermittent dosing, is required to suppress colon tumorigenesis in rats. *Toxicol Sci (suppl.)* **42**: 319

Barnes CJ, Cameron IL, Hardman WE and Lee M (1998*b*) NSAID modulation of colonic epithelial cell proliferation and apoptosis at intermediate biomarkers of induced rat colon cancer. *Br J Cancer* **77**: 573–580

Boolbol SK, Dannenberg AJ, Chadburn A, Martucci C, Guo XJ, Ramonetti JT, Abreu-Goris M, Newmark HL, Lipkin ML, Decosse JJ and Bertagnolli MM. (1996) Cyclooxygenase-2 overexpression and tumor formation are blocked by sulindac in a murine model of familial adenomatous polyposis. *Cancer Res* **56**: 2556–2560

Cameron IL, Ord VA, Hunter KE and Heitman DW (1990) Colon carcinogenesis: modulation of progression. In *Colon Cancer Cells*, Moyer MP and Poste G (eds), pp. 63–84. Academic Press: Orlando

Chiu C-C, Mcentee MF, and Whelan J (1997) Sulindac causes rapid regression of preexisting tumors in *Min*/+ mice independent of prostaglandin biosynthesis. *Cancer Res* **57**: 4267–4273

Craven PA and Derubertis FR (1992) Effects of aspirin on 1,2-dimethylhydrazine-induced colonic carcinogenesis. *Carcinogenesis* **13**: 541–546

Dubois RN, Radhika A, Reddy BS and Entingh AJ (1996) Increased cyclooxygenase-2 levels in carcinogen-induced rat colonic tumors. *Gastroenterology* **110**: 1259–1262

Eberhart CE, Coffey RJ, Radhika A, Giardiello FM, Ferrenbach S and Dubois RN (1994) Up-regulation of cyclooxygenase 2 gene expression in human colorectal adenomas and adenocarcinomas. *Gastroenterology* **107**: 1183–1188

Giardiello FM (1994) Sulindac and polyp regression. *Cancer Metastasis Rev* **13**: 279–283

Giovannucci E, Egan KM, Hunter DJ, Stampfer MJ, Colditz GA, Willett WC and Speizer FE (1995) Aspirin and the risk of colorectal cancer in women. *N Engl J Med* **333**: 609–614

Greenberg ER, Baron JA, Freeman Jr DH, Madel JS and Haile R (1993) Reduced risk of large-bowel adenomas among aspirin users. *J Natl Cancer Inst* **85**: 912–916

Jacoby RF, Marshall DJ, Newton MA, Novakovic K, Tutsch K, Cole CE, Lubet RA, Kelloff GJ, Verma A, Moser AR and Dove WF (1996) Chemoprevention of spontaneous intestinal adenomas in the Apc Min mouse model by the nonsteroidal anti-inflammatory drug piroxicam. *Cancer Res* **56**: 710–714

Kargman SL, O'Neill GP, Vickers PJ, Evans JF, Mancini JA and Jothy S (1995) Expression of prostaglandin G/H synthase-1 and -2 protein in human colon cancer. *Cancer Res* **55**: 2556–2559

Lecomte M, Laneuville O, Ji C, Dewitt DL and Smith WL (1994) Acetylation of human prostaglandin endoperoxide synthase-2 (cyclooxygenase-2) by aspirin. *J Biol Chem* **269**: 13207–13215

Lee M, Cryer B and Feldman M (1994) Dose effects of aspirin on gastric prostaglandins and stomach mucosal injury. *Ann Intern Med* **120**: 184–189

Marnett LJ (1992) Aspirin and the potential role of prostaglandins in colon cancer. *Cancer Res* **52**: 5575–5589

Meade EA, Smith WL and Dewitt DL (1993) Differential inhibition of prostagladin endoperoxide synthase (cyclooxygenase) isozymes by aspirin and other non-steroidal anti-inflammatory drugs. *J Biol Chem* **268**: 6610–6614

Morin PJ, Vogelstein B and Kinzler KW (1996) Apoptosis and APC in colorectal tumorigenesis. *Proc Natl Acad Sci USA* **93**: 7950–7954

Oshima M, Dinchuk JE, Kargman SL, Oshima H, Hancock B, Kwong E, Trzaskos JM, Evans JF and Taketo MM (1996) Suppression of intestinal polyposis in Apc delta 716 knockout mice by inhibition of cyclooxygenase 2 (COX-2). *Cell* **87**: 803–809

Pence BC, Dunn DM, Zhao C, Landers M and Wargovich MJ (1995) Chemopreventive effects of calcium but not aspirin supplementation in cholic acid-promoted carcinogenesis: correlation with intermediate endpoints. *Carcinogenesis* **16**: 757–765

Piazza GA, Rahm AL, Krutzsch M, Sperl G, Paranka NS, Gross PH, Brendel K, Burt RW, Alberts DS and Pamukcu R (1995) Antineoplastic drugs sulindac sulfide and sulfone inhibit cell growth by inducing apoptosis. *Cancer Res* **55**: 3110–3116

Rao CV, Rivenson A, Simi B, Zange E, Kelloff G, Steele V and Reddy BS (1995) Chemoprevention of colon carcinogenesis by sulindac, a nonsteroidal antiinflammatory agent. *Cancer Res* **55**: 1464–1472

Reddy BS, Rao CV, Rivenson A and Kelloff G (1993) Inhibitory effect of aspirin on azoxymethane-induced colon carcinogenesis in F344 rats. *Carcinogenesis* **14**: 1493–1497

© Cancer Research Campaign 1999 

Reddy BS, Rao CV and Seibert K (1996) Evaluation of cyclooxygenase-2 inhibitor for potential chemopreventive properties in colon carcinogenesis. *Cancer Res* **56**: 4566–4569

Sunter JP, Appleton DR, Wright NA and Watson AJ (1978) Pathological features of the colonic tumours induced in rats by the administration of 1,2-dimethylhydrazine. *Virchows Archiv B, Cell Pathol* **29**: 211–223

Thun MJ (1996) NSAID use and decreased risk of gastrointestinal cancers. *Gastroenterol Clin North Am* **25**: 333–348

Williams CS, Luongo C, Radhika A, Zhang T, Lamps LW, Nanney LB, Beauchamp ORD and Dubois RN (1996) Elevated cyclooxygenase-2 levels in Min mouse adenomas. *Gastroenterology* **111**: 1134–1340

Williams CS, Smalley W and Dubois RN (1997) Aspirin use and potential mechanisms for colorectal cancer prevention. *J Clin Invest* **100**: 1325–1329

*© Cancer Research Campaign 1999*

